# Distribution pattern of endophytic bacteria and fungi in tea plants

**DOI:** 10.3389/fmicb.2022.872034

**Published:** 2022-09-23

**Authors:** Haiyan Lin, Changwei Liu, Zhong Peng, Bin Tan, Kunbo Wang, Zhonghua Liu

**Affiliations:** ^1^Key Laboratory of Tea Science of Ministry of Education, Hunan Agricultural University, Changsha, China; ^2^National Research Center of Engineering and Technology for Utilization of Botanical Functional Ingredients, Hunan Agricultural University, Changsha, China; ^3^Co-Innovation Center of Education Ministry for Utilization of Botanical Functional Ingredients, Hunan Agricultural University, Changsha, China

**Keywords:** tea, leaf, endophytes, bacteria, fungi

## Abstract

Endophytes are critical for plant growth and health. Tea is an economically important crop in China. However, little is known about the distribution pattern and potential functions of endophytic communities in tea trees. In this study, two genotypes (BXZ and MF) cultivated under the same conditions were selected, and endophytic bacteria and fungi were analyzed through 16S rRNA and ITS high-throughput sequencing technologies, respectively. For endophytic bacteria, root tissues harbored the most diverse endophytes, followed by stems and old leaves, and new leaves possessed the lowest diversity. In contrast, old leave tissues harbored more diverse endophytic fungi than did root and stem tissues. Most of the dominant endophytes showed obvious cultivar and tissue preferences. Tissue type played a more important role in shaping community structure than did cultivar. Nevertheless, some endophytic bacterial groups, which mainly affiliated to *Chryseobacterium, Sphingomonas, Rhizobium, Morganella, Methylobacterium* and *Comamonadaceae*, could parasitize different tissues, and the average relative abundance of endophytic bacteria was as high as 72.57%. Some endophytic fungal populations, such as *Colletotrichum, Uwebraunia, Cladosporium, and Devriesia,* could also parasitize tea, and the relative abundance accounted for approximately 25.70–97.26%. The cooperative relationship between endophytic bacteria and fungi in the new leaves was stronger than that in the old leaves, which can better participate in the metabolism of tea material.

## Introduction

Endophytes are able to live inside plant tissues without inducing any apparent symptoms in their hosts (Borer et al., 2013). Numerous studies indicated that endophytes play important roles in plant disease control, secondary metabolites synthesis, plant growth regulation, and environmental resistance (Oukala et al., 2021). For this reason, endophytes have been receiving increasing attention from scientists since the latter part of the twentieth century. Fungi and bacteria are the most common microbes that exist as endophytes. It is reported that more than half of the isolated endophytes had different kinds of plant growth-promoting traits (Spaepen et al., 2009; Bulgarelli et al., 2012). These endophytes can promote plant growth by accelerating the availability of mineral nutrients, helping in the production of phytohormones, siderophores, and enzymes, and by activating systemic resistance against insect pests and pathogens in plants (Santoyo et al., 2016; Kushwaha et al., 2020). They can also regulate plant growth through improving nitrogen fixation, phosphate solubilization, siderophore, 1-aminocyclopropane-1-carboxylate (ACC) deaminase activity and indole-3-acetic acid (IAA) synthesis Santoyo. Meanwhile, endophytes can induce plant defenses through jasmonic acid, salicylic acid and ethylene pathways (Santoyo et al., 2016). Furthermore, it was also revealed that bacterial endophytes can improve salt tolerance by adjusting osmotic balance, ion homeostasis, phytohormone production, extracellular molecules and exopolysaccharides, which can be a more environmentally friendly and cost-effective solution to curtail the harmful effect of salinity on crop growth and yield (Ali et al., 2014; Singh et al., 2017). Likewise, some fungal endophytes, such as *Leptontidium*, *Phialocephala* and *Beauveria*, also affect plant growth and plant responses to pathogens, herbivores, and environmental changes (Hong et al., 2005; Porras-Alfaro and Bayman, 2011; Huang et al., 2020). All these studies suggested that endophytes would play important roles in helping plant growth and alleviating environmental stress on plant, but the distribution and interaction patterns between many host plants and endophytes remains unknown.

Tea (*Camellia sinensis*) is a perennial woody plant and an economically important crop in China (Li et al., 2022). The secondary metabolites of tea, such as tea polyphenols, theophylline, organic acids, can excite and relieve fatigue, detoxify and quench thirst, diuresis and improve eyesight, supplement nutrition, etc. (Bhuyan et al., 2013; Shan et al., 2018; Rothenberg and Zhang, 2019). Recently, a few researches has shown that there were also some endophytes colonized in tea plant, and these endophytes may also play important roles in helping plant growth and improving tea quality. For example, Yan et al. (2018) isolated 274 bacterial isolates from two tea cultivars, these endophytic bacterial mainly affiliated to Proteobacteria, Firmicutes and Bacteroidetes. Some of the endophytic bacteria appeared plant-growth promoting (PGP) traits, such as nitrogen fixation, P-solubilization, siderophore, IAA production or ACC deaminase. Sun et al. (2019) isolated an endophytic bacterial strain (identified as *Luteibacter* spp.) with strong biocatalytic activity for converting both glutamine and ethylamine to theanine. Likewise, endophytic fungus *Colletotrichum gloeosporioides* isolated from healthy tea plant tissues also showed a strong inhibitory activity on tea plant pathogens of *Pestalotiopsis theae* and *Colletotrichum camelliae*, and the inhibitory mechanism may attribute to the fungus’ high efficient chitinase and protease (Rabha et al., 2014). However, these few studies mainly focused on the functions of endophytic strains based on traditional isolation, which would extremely limit our comprehensive understanding of the distribution pattern of endophytes in tea plant.

In this study, two tea plant varieties under the same environmental conditions and planting management measures were selected as materials, and the characteristics of endophytic populations in roots, stems, old leaves and new leaves were systematically compared and analyzed through 16S and high-throughput sequencing technology. The purpose is to explore the population distribution characteristics of the quality endophytes, the functional activity of dominant populations and their interaction with environmental microorganisms and to clarify the characteristics of the distribution of the endophytic bacterial population in tea plants. Therefore, exploring the population distribution characteristics and functions of tea tree endophytes can lay a foundation for the evaluation of the influence of endophytes on the formation of tea quality and provide a basis for further research and development of tea tree endophytic resources.

## Materials and methods

### Sampling of tea plant tissue

The experiment was carried out in the Chang’an station of Hunan Agricultural University. The type of soil was red soil. The physical and chemical properties of the soil were as follows: organic carbon 0.95 g·kg^−1^, total nitrogen 0.97 g·kg^−1^, and total phosphorus 0.35 g·kg^−1^. Total potassium 1.52 g kg^−1^, available potassium 92 mg kg^−1^, available phosphorus 26.6 mg kg^−1^, alkaline hydrolysis nitrogen 112 mg kg^−1^, available iron 138 mg kg^−1^, available manganese 15.4 mg kg^−1^, available copper 1.14 mg kg^−1^, available zinc 1.57 mg kg^−1^, pH 3.87. Two typical cultivars of tea plants (BXZ and MF) with 5 years planting history under the same management practices (such as fertilization) were selected in 2017. For each cultivar., 15 healthy tea plants with the uniform growth were selected, and for each plant, the third and sixth leaves of each plant were sampled as new and old leaves, respectively. Roots and stems were simultaneously collected and washed off soil particles with water. All the tissues were surface-sterilized by successively submersing in 75% ethyl alcohol for 3 min, 1.2% sodium hypochlorite for 3 min, and then 75% ethyl alcohol for 1 min, followed by five rinses with sterilized deionized water. The final rinse water was used to verify the sterility of root surfaces by both PCR amplification of 16S rRNA gene and plate cultivation method (Seghers et al., 2004). The verified tissue samples were immediately frozen in liquid nitrogen and stored at-80°C for molecular analysis.

### DNA extraction and endophytic bacterial sequencing

A Fast DNA Spin Kit (Fast DNA Spin Kit for Soil, MP) was used for DNA extraction from tea plants. The concentration and purity of DNA were determined using a NanodropND-1,000 (NanoDrop Technologies, Delaware, United States). After DNA concentration was diluted to 30 ng uL-1 with nuclease-free water, all the samples were performed first PCR to amplify bacterial 16S rRNA (V5-V7) with 799F/1492R primers (Bulgarelli et al., 2012). The reaction solution consisted 1 μl (10 μmol L − 1) of each forward and reverse primer, 2 μl of template DNA, 25 μl 2 × Power Taq Master Mix (Tiangen, Beijing, China), and 21μLnuclease-free water. PCR reaction was performed with a Mastercycler pro gradient PCR Cycler (Eppendorf AG, Hamburg, German) as follows: 94°C for 2 min, 35 cycles of 94°C for 30 s, 55°C for 30 s,72°C for 45 s, and with a final extension of 72°C for 6 min. The amplification of genes was checked using gel electrophoresis (1.5% agarose) and the band of approximately 700 bp was excised and purified using an AxyPrep DNA kit (Axygen, California, United States). The purified PCR product from the first PCR reaction was used as templates for the second PCR amplification (using same conditions) with the primer set 799F/1193R (Lundberg et al., 2012) that comprised an eight-base barcoded at the 5′ end. For the endophytic fungi, primer set of ITS1F/ITS2R was used to amplify ITS genes according to Arnold et al. (2003). The PCR amplicons were gel purified as described above and quantified using a NanodropND-1,000 spectrophotometer. These purified products were pooled in equimolar aliquots and sent for paired-end sequencing on an Illumina Miseq PE300 platform (Shanghai MajorbioBioPharm Technology Co., Ltd., Shanghai, China).

### Bioinformatics and statistical analysis

The obtained raw 16S rRNA gene sequences were quality filtered, assembled, de-multiplexed and assigned to individual samples using Quantitative Insights into Microbial Ecology (QIIME) pipeline (version 1.9.0). Briefly, sequences were discarded if they contained any ambiguous base, had more than two mismatches to the primers, one mismatch to the barcode sequence, or a minimum sequence length of 200 bp or average quality score of 20. After filtering and chimera removal, the operational taxonomic units (OTUs) picking was performed using USEARCH at 97% sequence identity. The singletons were filtered, and subsequently, taxonomic annotation of the representative 16S rRNA and ITS sequences was assigned based on the Greengenes database (version 13.5) and unite database (version 7.0) using RDP classifier Bayes algorithm with a confidence threshold of 70% (Goyer et al., 2022). After removing the sequences related to chloroplast or mitochondria, number of OTUs, Shannon, and Chao1 indexes were calculated based on the OTU table that was unified to 9,146 and 36,037 sequences (the minimum number of sample sequences) per sample for 16S rRNA and ITS genes, respectively. Hierarchical cluster diagram and principal coordinates analysis (PCoA) were performed to display the similarities or differences in the community compositions between samples. In order to further identify endophytic bacterial/fungal taxa detected in tea trees that are omnipresent across all cultivar and tissue types or locally resident in specific cultivar or tissue types, a Venn diagram was used to split the overall community into two general categories: “common” - OTUs detected in all samples, “specific”—OTUs found only in the specific tissue samples from a specific cultivar. One-way analysis of variance (ANOVA) was used to detect the significant difference between the properties of soil and plant with SPSS software (SPSS Inc., Chicago, United States). The Duncan’s multiple range was used for detecting significant difference among treatments, and the statistical significance was determined at *p* < 0.05 and highly significant difference at *p* < 0.01.

The correlation between the endophyte populations in the sample was analyzed on the online tool of Majorbio Cloud Platform with default parameters.^1^

## Results

### Community diversity of endophytic bacteria and fungi in tea trees

**Figure 1:**
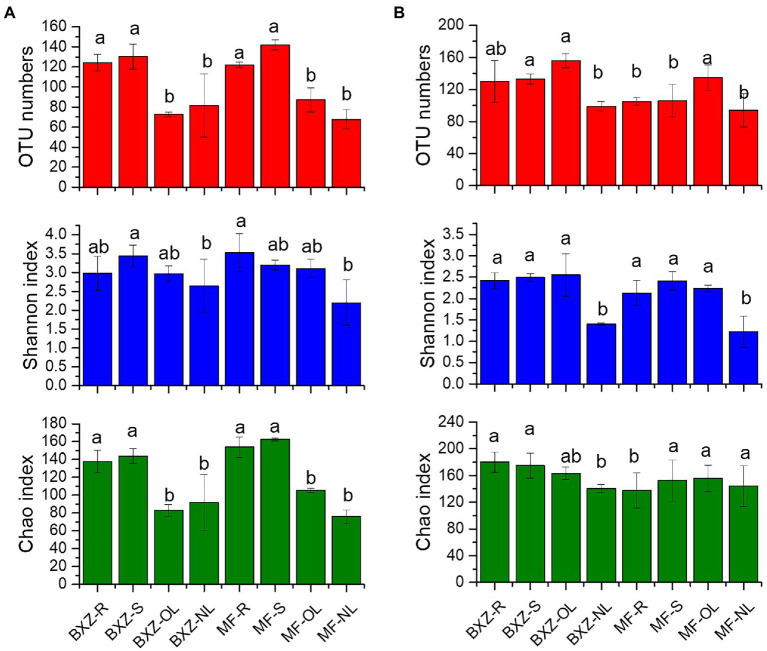
Diversity of endophytic bacteria **(A)** and fungi **(B)** in tea trees. BXZ_R, BXZ_S, BXZ_OL 556 and BXZ_NL represented root, stem, old leave and new leave samples of BXZ cultivar, while MF_R, 557 MF_S, MF_OL and MF_NL represented root, stem, old leave and new leave samples for MF cultivar. Different letter indicates (a) BXZ tea tree α diversity and (b) MF tea tree α diversity.

After quality control and chloroplast or mitochondrial sequences removal, a total of 530,608 bacterial sequences and 2,442,189 fungal sequences were obtained from 24 samples. These sequences were assigned to 636 bacterial OTUs and 718 fungal OTUs, respectively. In comparison, the OTU numbers and Chao index of endophytic bacteria in roots and stems were significantly higher than that in leaves for both BXZ and MF (*p* < 0.05), while no significant differences (*p* > 0.05) were detected between roots and stems, and old and new leaves. But for the shannon index, although similar pattern were observed, only MF-root and BXZ-stem samples appeared significantly higher values than new leaves, while other tissues showed no significant differences (Figure 1A).However, no significant differences in bacterial diversity were detected between two cultivars for specific tissue (Figure 1A). Unlike bacteria, for the endophytic fungi, it was detected that the new leaves generally showed the lowest OTU numbers and Shannon index for both BXZ and MF, while similar fungal diversity was detected in root, stem and old leaves, except for the old leaves showed higher OTU numbers (Figure 1B). Furthermore, significant differences in fungal diversity were also detected between two cultivars for specific tissues, e.g., OTU numbers in stem, Chao1 in root (*p* < 0.05).

### Community structures of endophytic bacteria and fungi in tea trees

**Figure 2:**
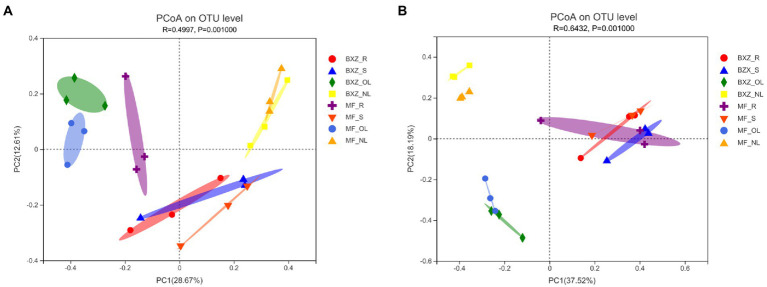
PCoA analysis of community structures of endophytic bacteria **(A)** and fungi **(B)** in tea trees. BXZ_R, BXZ_S, BXZ_OL and BXZ_NL represented root, stem, old leave and new leave samples of BXZ cultivar., while MF_R, MF_S, MF_OL and MF_NL represented root, stem, old leave and new leave samples for MF cultivar.

From the PCoA plots (Figure 2), it was detected that although cultivar also induced some changes in their community structure for specific tissue types, all the samples separately clustered according to tissue types, suggesting that the tissue type may play a more important role in shaping endophytic bacterial community structure than did cultivar (Figure 2A). Among the four tissue types, the bacterial community in root and stem samples clustered closer while that in old and new leaves was clearly apart. These results suggested that the root and stem may share relative similar community structure of endophytic bacteria and fungi, while old or new leaves may harbor distinct endophytic community structures (*p* < 0.01; Figure 2A). Generally, the distribution pattern of the fungal community in tea trees were quite similar with that of bacteria (Figure 2B), which were also characterized by separately clustered according to tissue types with some modification induced by cultivar.

**Figure 3:**
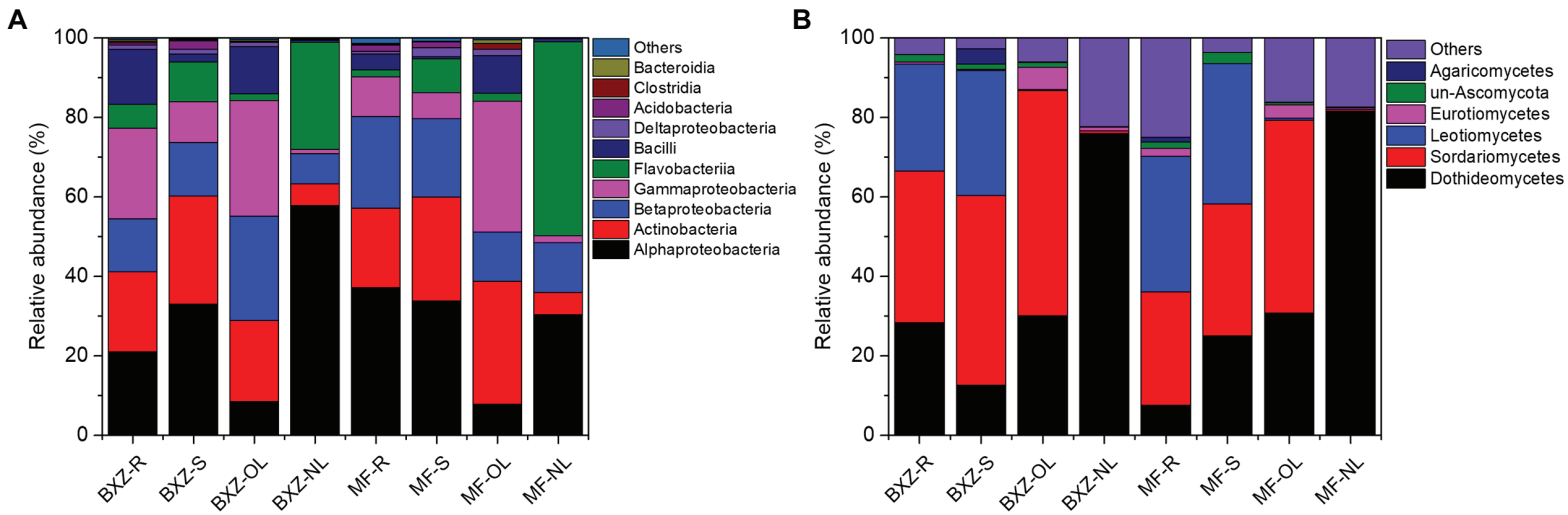
Taxonomic analysis of the endophytic bacterial **(A)** and fungal **(B)** communities at class level. BXZ_R, BXZ_S, BXZ_OL and BXZ_NL represented root, stem, old leave and new leave samples of BXZ cultivar., while MF_R, MF_S, MF_OL and MF_NL represented root, stem, old leave and new leave samples for MF cultivar.

The taxonomic analysis further revealed that the endophytic bacteria and fungi differed markedly among the tissue types and cultivars (Figure 3). It was observed that, on average, more than 90% of observed endophytic bacteria were affiliated to six bacterial classes, including Alpha-, Beta-and Gamma-proteobacteria, Actinobacteria, Flavobacteriia and Bacilli (Figure 3A). However, the relative abundance of these taxa varied among different tissue types as well as cultivars (Figure 3A). For example, new leaves in both BXZ and MF were predominated by Alphaproteobacteria and Flavobacteriia, which totally account for 84.8 and 79.2% of the total endophytic bacterial population, respectively. However, the relative abundances of these two dominant bacterial groups were decreased to 10.2 and 9.8% in old leaves of BXZ and MF, respectively. Instead, the minor groups Actinobacteria and Gammaproteobacteria in new leaves (average total proportion 3.2–3.6%), were significantly increased to be predominant endophytic bacterial groups (averagely 24.7–32.0%) in old leaves for both BXZ and MF. The root and stem samples generally shared similar community structure of endophytic bacteria for both cultivars, and the relative abundance of the dominant groups described above were generally in between new and old leaves. Finer taxonomic division at genus level (Supplementary Figure S1A) showed that members of *Morganella, Acidovorax,* and *Rhizobium* were dominant populations in roots and stems. But in new leaves, approximately 70% of the bacteria belonged to the four major bacterial genera *Chryseobacterium, Sphingomonas, Rhizobium* and *Methylobacterium*. The population composition of old leaves was more complex than that of new leaves. *Arsenophonus, Corynebacterium, Actinomyces, Pseudomonas* and other taxa were dominant populations in old leaves, but the amounts in other tissues were relatively low or even undetectable.

Likewise, most of the dominant endophytic fungal taxa also appeared tissue preference(Figure 3B). For example, the roots and stems were dominated by fungal classes of Dothideomycetes, Sordariomycetes and Leotiomycetes, which totally took account 87.0–93.5% of the overall community of endophytic fungi. Dothideomycetes and Sordariomycetes were still dominant fungal group in old leaves, but the relative abundance of Leotiomycetes were significantly decreased by 26.7–34.8%, which only represented 0.3 and 0.5% of the total fungal population in the old leaves of BXZ and MF, respectively. With regards to new leaves, it was interestingly found that approximately 76.0 and 81.6% of the endophytic fungal community belonged to Dothideomycetes, while other taxa only took account minor proportion. When analysis at genus level (Supplementary Figure S1B), it was found that approximately 38.0–90.3% were unclassified taxa. Of the classified taxa, *Colletotrichum* (4.8–9.7%), *Scleropezicula* (2.2–17.2%) and *Devriesia* (1.1–2.7%) were dominant fungal groups in roots and stems across two cultivars with proportion > 1%. With respected to old leaves, the proportion of *Colletotrichum*, *Cladosporium* and *Aspergillus* were significantly increased to 36.2–41.4%, 4.6–6.0% and 1.9–2.1%, respectively. Instead, the dominant group in root and stems, such as *Scleropezicula* and *Devriesia*, were decreased to be minor groups with relative abundance less than 0.1%. Compared to old leaves, the proportion of *Colletotrichum* was sharply decreased to 0.05–0.16% in new leaves, while *Uwebraunia* remarkably increased to be the predominant taxa with relative abundance 8.2–49.6%.

### Partitioning of endophytic bacterial and fungal community

**Figure 4:**
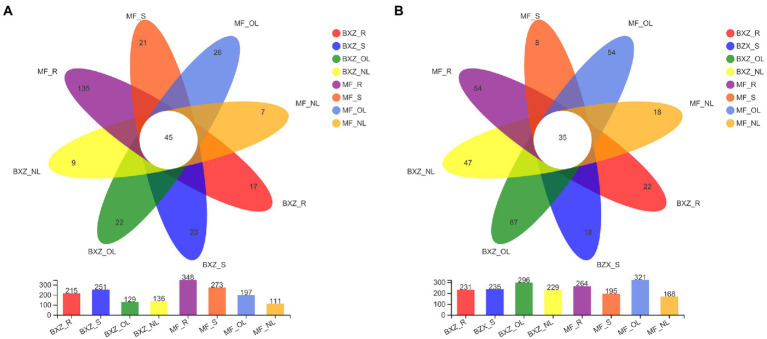
Common, tissue/cultivar-specific bacterial **(A)** and fungal **(B)** OTUs in tea tree. BXZ_R, BXZ_S, BXZ_OL and BXZ_NL represented root, stem, old leave and new leave samples of BXZ cultivar., while MF_R, MF_S, MF_OL and MF_NL represented root, stem, old leave and new leave samples for MF cultivar.

The distribution of endophytic bacterial and fungal OTUs among samples could be visualized in the Venn diagrams (Figure 4). It was detected that 45 bacterial OTUs were shared by all tissue samples across two cultivars; these OTUs formed 25.0% on average (12.9–40.5%) of the total OTU numbers from a given tissue type. By comparison, the factions of common OTUs in leaves were higher than that in roots and stems across two cultivars (Figure 4A). However, when examining the relative abundance of the common OTUs in a specific tissue sample, they represented, on average, 72.6% of the total bacterial sequence reads, with the highest proportion of 95.9% in new leaves, followed by stems (81.2%), and the lower in old leaves and root samples at 56.0 and 54.3%, respectively (Table 1).

**Table 1:** Proportions of common and specific taxa in total OTU numbers and population sizes of endophytic bacteria and fungi in tea trees.

	Proportion of OTU numbers (%)				Proportion of sequence reads (%)			
	Bacteria		Fungi		Bacteria		Fungi	
	**Common**	**Specific**	**Common**	**Specific**	**Common**	**Specific**	**Common**	**Specific**
BXZ-R	20.93	7.91	15.15	9.52	72.64	0.75	39.92	0.07
BXZ-S	17.93	9.16	14.89	7.66	82.29	0.68	29.07	0.05
BXZ-OL	34.88	17.05	11.82	29.39	57.58	4.45	84.37	2.16
BXZ-NL	33.09	6.62	15.28	20.52	94.05	0.30	94.14	0.22
MF-R	12.93	38.79	13.26	20.45	35.90	6.83	25.70	7.49
MF-S	16.48	7.69	17.95	4.10	80.07	0.46	34.65	0.01
MF-OL	22.84	13.20	10.90	16.82	60.38	1.96	85.14	0.88
MF-NL	40.54	6.31	20.83	10.71	97.68	0.24	97.26	0.07

BXZ_R, BXZ_S, BXZ_OL and BXZ_NL represented root, stem, old leave and new leave samples of BXZ cultivar. while MF_R, MF_S, MF_OL and MF_NL represented root, stem, old leave and new leave samples for MF cultivar.

For the endophytic fungal communities, it was detected that 35 OTUs were assigned as common community (Figure 4B). On average, these common OTUs represented 15.0% (10.9–20.8%) of the total OTU numbers in fungal community in a specific tissue sample, with the highest proportion of 15.3 and 20.8% in new leaves of BXZ and MF, respectively, and the lowest in old leaves at 11.8 and 10.9%, respectively. However, the relative abundance of these common communities in the total fungal population of a specific sample varied among tissue types. The average relative abundance of common groups was approximately 94.1–97.3% in new leaves, and 84.4–85.1% in old leaves, which were significantly higher than that in root and stem samples (32.8 and 31.9% on average).

When concerning the bacterial and fungal OTUs occurred in only specific tissue and cultivar., it was revealed that the proportions of the specific bacterial OTUs were generally lower than the common communities, which took account 13.3% of the total OTU numbers in individual sample, on average. The lowest number of OTUs assigned to the specific category was observed for new leaves in both BXZ and MF, representing 6.62 and 6.31% of their total OTU numbers, respectively. However, their proportions in old leaves were significantly increased to 17.05 and 13.20%, respectively. The proportion of specific OTUs in roots varied significantly with cultivar., with 7.91% in BXZ but 38.79% in MF. Unlike endophytic bacteria, the lowest specific fungal OTU number proportion were observed in stems, the percentage in BXZ and MF were 7.66 and 4.10%, respectively. The highest proportion in BXZ occurred in old leaves (29.39%), but in MF, it was changed to roots (20.45%). However, although the specific bacterial or fungal endophytic taxa averagely represented 13.34–14.90% in the total OTU pools, their relative abundance within the endophytic population were very low, on average, only 1.96 and 1.37% of the total bacterial and fungal reads in a specific sample, respectively.

### Taxonomy analysis of common and tissue/cultivar specific community of endophytic bacteria and fungi

**Figure 5:**
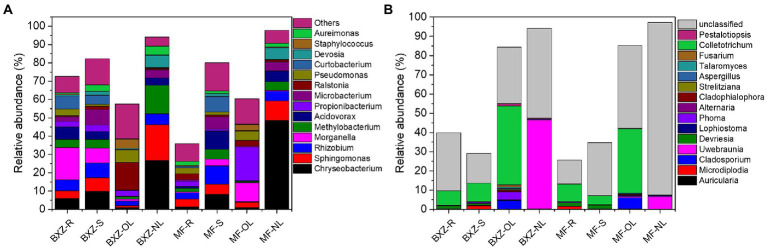
Common bacterial **(A)** and fungal **(B)** endophytic communities that shared by all tissues across two cultivars (genus level). BXZ_R, BXZ_S, BXZ_OL and BXZ_NL represented root, stem, old leave and new leave samples of BXZ cultivar., while MF_R, MF_S, MF_OL and MF_NL represented root, stem, old leave and new leave samples for MF cultivar.

Taxonomy analysis showed that the common bacterial OTUs were mainly affiliated to 7 bacterial classes of Alpha-, Beta-and Gamma-proteobacteria, Actinobacteria, Flavobacteriia, Bacilli (Figure 5A), and 31 bacterial genera, such as *Chryseobacterium, Sphingomonas, Rhizobium, Morganella, Methylobacterium* and *Comamonadaceae* (Figure 5A). The common fungal taxa were mainly distributed in 4 fungal classes (*Dothideomycetes, Eurotiomycetes, Sordariomycetes, Agaricomycetes,* etc.), 12 fungal orders (*Capnodiales, Pleosporales, Eurotiales,* etc.), and 15 fungal genera (*Colletotrichum, Uwebraunia, Cladosporium,* etc.; Figure 5B). However, although these common taxa diversely distributed in various tissue types across cultivar., their relative abundance varied significantly among samples. For example, new leaves harbored the highest proportion of common communities affiliated to bacterial genera of *Chryseobacterium* and *Devosia*, followed by stems and roots, and the old leaves possessed the lowest proportion. The relative abundance of *Curtobacterium* in root and stem samples were significantly higher than that in old and new leaves (*p* < 0.05; Figure 5A). For the fungal community, old leaves had between 2.7–6.0 times higher proportion of *Colletotrichum* than root and stem samples, and the proportion of this taxa sharply decreased from 33.3–41.0% in old leaves to 0.05–0.13% in new leaves. In contrast, the new leaves possessed the highest proportion of *Uwebraunia* (46.3% in BXZ and 6.5% in MF), followed by old leaves (0.3 and 0.5%), and the proportion in roots and stems was very low (less than 0.02%; Figure 5B).

**Figure 6:**
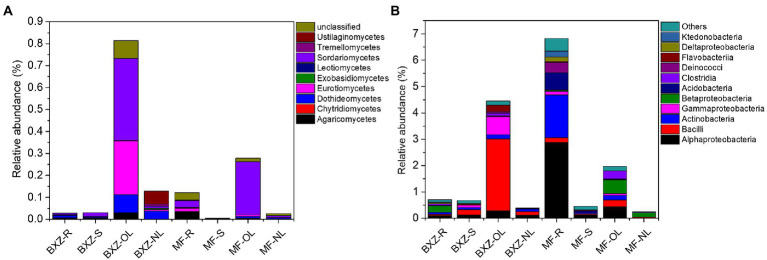
Specific bacterial **(A)** and fungal **(B)** endophytic communities that only occurred in specific tissue/cultivar (class level). BXZ_R, BXZ_S, BXZ_OL and BXZ_NL represented root, stem, old leave and new leave samples of BXZ cultivar. while MF_R, MF_S, MF_OL and MF_NL represented root, stem, old leave and new leave samples for MF cultivar.

In comparison with the common community, the specific communities of endophytic bacteria and fungi were diversely distributed in 31 bacterial and 11 fungal classes, and 96 bacterial and 81 fungal genera, respectively. Furthermore, the taxonomy categories of both bacterial and fungal OTUs identified as specific were also closely related to tissue and cultivar types (Figure 6). For example, of the total specific bacterial OTUs identified in old leaves of BXZ, about 61.09% of the community related to the class Bacilli (e.g., genera *Alicyclobacillus*) and other specific OTUs belonging to Alpha-and Gamma-proteobacteria (*Cardiobacterium, Nevskia*), Flavobacteriia (*Flavobacterium, Candidatus_Uzinura*), were observed in lower proportions (> 0.2%). In old leaves of MF, bacterial classes of Alpha-and Beta-proteobacteria, Clostridia, and Bacilli, and bacterial genera of Prevotella, Paenibacillus, Eubacterium, Corynebacterium, and Alloprevotella were the abundant specific groups with relative abundance ranged from 0.14 to 0.51%. Whereas in the root samples of MF, Alpha-proteobacteria (e.g., *Roseomonas, Sphingobium, Inquilinus*), Actinobacteria (*Catenulispora, Actinospica, Acidothermus, Nocardia*, etc.), and Acidobacteria (*Bryobacter, Candidatus_Solibacter*) were predominant groups with relative abundance >0.5%, which totally accounted 75.72% of the total specific community. For the specific fungal community (Figure 6B), it was detected that the old leaves in BXZ were dominated by fungal classes of Sordariomycetes (45.86% of the total specific fungal community), Eurotiomycetes (29.97%), and fungal genera of *Acremonium*, *Talaromyces*, *Rachicladosporium, Knufia*, and *Verticillium*. While approximately 86.71% of the specific fungal community in MF old leaves were affiliated to *Sordariomycetes*, including fungal genera of *Lecanicillium, Myrmecridium*, and *Rosellinia*.

### Analysis of the interaction between endophytic bacterial and fungal populations

The relationship between the endophytic bacteria and fungi in the different tissues of BXZ and MF was analyzed through the species correlation network.

**Figure 7:**
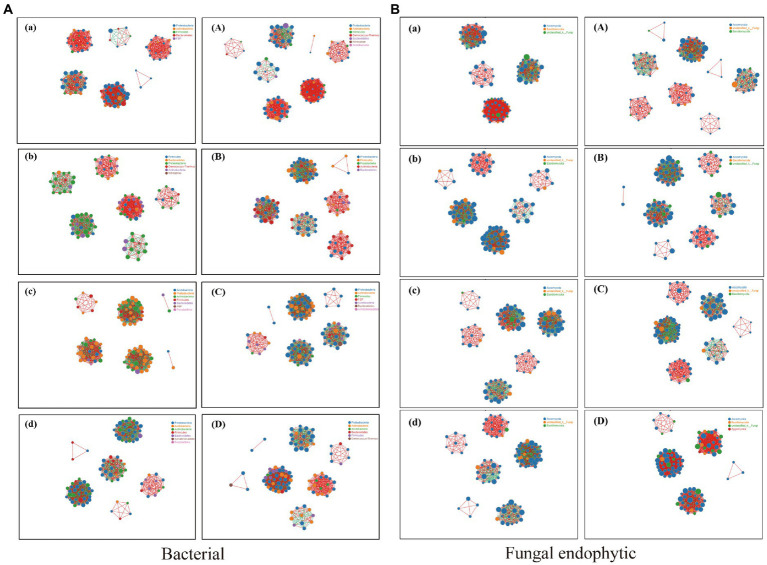
Network analysis showing the correlations between bacterial and fungal endophytic communities. (The figure shows the species with Spearman coefficient > 0.8 and *p* < 0.01 by default; the size of the node in the figure indicates the species abundance, and different colors indicate different species; the color of the line indicates a positive and negative correlation, and red indicates a positive correlation. Green indicates a negative correlation; the thickness of the line indicates the size of the Pearson correlation coefficient. The thicker the line, the higher the correlation between species; the more lines, the closer the relationship between the species and other species.) **(A)** Network analysis of correlation between BXZ and endophytic bacteria. **(B)** Network analysis of correlation between MF and endophytic bacteria. (a) BXZ new leaf; (b) BXZ old leaf; (c) BXZ stem; (d) BXZ root system; (A) MF new leaf; (B) MF old leaves; (C) MF stem; (D) MF root system.

There were significant interactions between different species of endophytic bacteria and fungi among different tissues. The degree of correlation was significantly different, which was represented by root > stem > old leaves > new leaves (Figure 7). The number of bacterial and fungal populations (a positive correlation) in the new leaves was much higher than that in the old leaves. Compared with new leaves, the number of populations with a negative correlation in old leaves increased significantly. The correlation between bacterial populations in roots and stems was more complex, mainly manifested in the existence of positive and negative correlations between species.

## Discussion

In this study, 16S rRNA and ITS high-throughput sequencing analysis were performed on the endophytes of BXZ and MF under the same cultivation management, and the difference and contrast analysis of the endophyte population composition and diversity in different tissues were carried out. These findings indicate that the endophytes in different tissues of different varieties are significantly different and are represented by roots > stems > old leaves > new leaves.

The endophyte community structure within the plant is dynamic and is influenced by abiotic and biotic factors such as soil conditions, biogeography, plant species, microbe-microbe interactions and plant-microbe interactions, both at local and larger scales (García-Guzmán and Heil, 2014). There is a long-term coevolution relationship between endophytes and tea. We found 636 species of endophytic bacteria of tea plants in this study, belonging to 20 bacterial phyla, 35 bacterial classes, 81 bacterial orders, 169 bacterial families, and 308 bacterial genera.

Endophytes are rich in plant tissues and play important roles in plant-microbial interactions and plant-growth regulation (Hardoim et al., 2008; Hacquard and Schadt, 2015; Wani et al., 2015). The bacterial groups of different tea plant species under the same cultivation conditions may be determined by different root exudates, different signaling molecules, etc. (Shyam et al., 2010; Frank et al., 2017). The root system was in direct contact with the soil, and the bacteria in the soil had more opportunities to infect the root system. Therefore, the diversity of endophytes in the roots was much higher than that in other tissues. Generally, host plants and rhizosphere endophytes are mostly symbiotic relationships. Previous studies have found that *Azoarcus, Burkholderia, Gluconobacter, Herbaspirillum, Klebsiella, Plantoea and Rahnella* and other bacterial genera can be detected in many plants (Dudeja et al., 2021). These bacteria can help host plants relieve nutrient stress by fixing nitrogen and secreting growth hormone under relatively infertile conditions (Dudeja et al., 2021). Some endophytic bacterial populations in plants, such as *Bacillus, Enterobacter, Pseudomonas, Streptomyces,* etc., can help host plants resist pathogenic bacteria and drought, salinity and other biological and abiotic stresses (Ali et al., 2014; Mukherjee, 2014; Naveed et al., 2014). We found that some bacterial genera, such as *Chryseobacterium, Sphingomonas, Rhizobium, Morganella, Methylobacterium Propionibacterium,* etc. were dominant bacterial populations coexisting in different tissues of BXZ and MF. It has been proven that bacterial populations such as *Chryseobacterium* have strong environmental tolerance and can adapt to oligotrophic and hypoxic environments (Kampfer et al., 2003; Liu et al., 2010).

The distribution of endophytic bacteria in tea trees showed an obvious alternating pattern during the maturation of tea leaves (Arnold et al., 2003; Suryanarayanan and Thennarasan, 2004). The types and numbers of endophytic bacteria gradually increased as the leaves matured. For example, the contents of *Arsenophonus, Corynebacterium, Actinomyces, Pseudomonas*, etc. in old leaves had significantly increased when compared with new leaves. Is the genetic background or growth characteristics of the tea plant itself or the external environmental conditions responsible for the differences in the distribution of endophytic bacteria populations? The answers to these questions need to be further explored and studied. The endophytic bacteria in the new leaves had a positive correlation, and they participated in the formation of tea metabolites together and jointly promoted the growth of the new leaves. However, the endophytic bacteria in the old leaves mainly showed a competitive relationship. To satisfy their own growth, the endophytic bacterial populations had a competitive relationship for nutrients.

Some endophytic bacterial populations could be parasitic in different tissues or varieties (Wei et al., 2018). Our results indicated that these endophytic bacterial populations, including *Chryseobacterium, Sphingomonas, Rhizobium, Morganella, Methylobacterium and Comamonadaceae*, that exist in different varieties and under different cultivation conditions may be common endophytic bacterial populations in tea varieties. The abundance, distribution and diversity of endophytes may be influenced by the genotypes and tissue types of tea plants and the length of plant growth. In this study, among the 636 different species of bacteria obtained from BXZ and MF planted in the same cultivation environment, only 45 species were shared. The microenvironment in different species or tissues affects endophytic bacterial survival. Some endophytic bacteria preferred the microenvironment in the leaf, and the abundance in the leaf was higher than that in other tissues. Previous studies have shown that environmental requirements, such as nutrient status, pH, oxygen concentration, etc. had a great influence on the growth of tea microorganisms. The difference in habitat conditions can directly affect their living conditions and functional activity (Horner-Devine et al., 2007; Fierer and Ladau, 2012; Hazard et al., 2013). Factors such as genetic background and growth characteristics will lead to certain differences in the physical structure, chemical composition and nutritional components of different tissues, which will cause changes in the composition and structure of the tea plant bacterial population. The differences in the endophytic bacterial population structure of BXZ and MF may be related to the genetic characteristics of tea plants.

There was also a long-term coevolutionary relationship between endophytic fungi and plants. Some endophytic fungi have developed into an inseparable part of the plant (Osono and Mori, 2003). The distribution of endophytic fungi in tea plants had obvious specificity among different tissues. We found an obvious trend of old leaves > new leaves > roots > stems according to the types and quantity of endophytic fungi. Different tissues had different chances of contacting fungal spores floating in the air and fungi in the soil during the growth process. These endophytic fungi could produce metabolites with the same or similar chemical structure as the host (Zeng et al., 2000). Tea plants might recruit and colonize specific endophytic fungal groups based on their genetic and biochemical characteristics to maintain their own healthy growth (Su et al., 2010). However, which endophytic fungal groups are closely related to the genetics of tea trees and whether these endophytic fungal groups have a certain internal connection between the healthy growth of tea trees and tea quality are still unclear.

In addition to a large number of bacterial populations, there are also a large number of fungi in the soil, which greatly enriches the diversity of endophytic fungi in the root system. Hu et al. (2013) isolated 7 genera and 12 species of endophytic fungi from the tea roots of 10-year-old “Longjing long leaves.” The 35 strains of endophytic fungi isolated by Agusta et al. (2005) belonged to *Fusarium, Penicillium, Schizophyllum and Diaporthe*. Studies have also found that the infection of tea plant endophytic fungi may be related to the number of stomata on the leaves. As the leaves mature, the number of stomata gradually increases (Xie et al., 2006; Xie, 2007). The composition of endophytic fungi in leaves at different developmental stages may be related to the internal and external structure and chemical factors of the leaves. How tea biochemistry affects the succession of endophytic fungi and whether endophytes have an impact on tea biochemistry require further research.

Endophytic fungi can survive in different endophytic microenvironments of tea plants. Taxonomic analysis found that most of the dominant fungal populations coexisting in different parts of different tea plant species belong to the genera of *Colletotrichum, Uwebraunia, Cladosporium*, and *Devriesia*. These ubiquitous endophytic fungi could live widely in plants; they changed in the microenvironment and affected their survival status. Most fungi had specific habitat requirements, including nutrient status, pH, oxygen concentration, etc. The differences in habitat conditions could directly affect their survival status and functional activity. The different varieties of tea plants used in this study had certain differences in the physical structure, chemical composition, and nutrient conditions of their roots, stems and leaves due to the genetic background and growth characteristics. The composition and structure of the fungal population in the organ would be different.

The endophytic fungal populations had a similar bacterial trend, the new leaves were more in the form of a positive correlation, and they participated in the material metabolism of tea through mutual help. The nutrients in the old leaves were reduced, and the endophytic fungal populations competed for nutrients to satisfy self-growth, which led to a significant reduction in the cooperative relationship between the endophytic fungal populations.

## Conclusion

Tea plant harbored high diversity of endophytic bacteria and fungi. Tissue type played a more important role in shaping community structure and diversity of endophytic bacteria and fungi than did cultivar. Roots harbored the most diverse endophytic bacteria while the new leaves possessed the lowest diversity. Whereas for endophytic fungi, old leave displayed higher diversity than did root and stem tissues. Most of the dominant endophytes showed obvious cultivar and tissue preferences. Nevertheless, some core endophytic groups with high abundance, such as bacterial general of *Chryseobacterium, Sphingomonas, Rhizobium, Morganella, Methylobacterium* and *Comamonadaceae,* and fungal groups of *Colletotrichum, Uwebraunia, Cladosporium*, and *Devriesia*, could parasitize different tissues. At the same time, the distinctive characters of individual tissue/cultivar resulted in highly diverse specific communities that appear to be restricted to specific environments.

## Data availability statement

The data presented in the study are deposited in the NCBI repository, accession number PRJNA861887.

## Author contributions

HL, KW, and ZL conceived and designed research. HL, CL, and ZP conducted the experiments. HL, ZP, and BT analyzed the data. HL wrote the manuscript. KW and ZL revised and perfected the manuscript. All authors contributed to the article and approved the submitted version.

## Funding

This research was funded by the National Key R&D Program of China (Grant no. 2018YFC1604403). Open research project of Hunan Key Laboratory for crop germplasm innovation and resource utilization Key scientific research project of the Department of education 21A0130.

## Conflict of interest

The authors declare that the research was conducted in the absence of any commercial or financial relationships that could be construed as a potential conflict of interest.

## Publisher’s note

All claims expressed in this article are solely those of the authors and do not necessarily represent those of their affiliated organizations, or those of the publisher, the editors and the reviewers. Any product that may be evaluated in this article, or claim that may be made by its manufacturer, is not guaranteed or endorsed by the publisher.

## Footnotes

^1^ https://cloud.majorbio.com/page/tools/
